# P-1127. Outcomes of Guideline Discordant Tracheal Aspirate Cultures in the Pediatric ICU

**DOI:** 10.1093/ofid/ofae631.1314

**Published:** 2025-01-29

**Authors:** Kerry Iles, Zachary Picciarelli, Glenn J Rapsinski, Zachary Aldewereld

**Affiliations:** UPMC Children's Hospital of Pittsburgh, Pittsburgh, Pennsylvania; UPMC Children's Hospital of Pittsburgh, Pittsburgh, Pennsylvania; Children's Hospital of Pittsburgh of UPMC, Pittsburgh, Pennsylvania; University of Pittsburgh Medical Center, Pittsburgh, Pennsylvania

## Abstract

**Background:**

Life-saving mechanical ventilation is associated with risk of ventilator-associated infections, though diagnosis of such infections can be difficult. Diagnostic respiratory cultures are often obtained via tracheal aspirate. These cultures can be misleading due to the non-sterile nature of the respiratory tract and the potential for chronic colonization of endotracheal and tracheostomy tubes by pathogenic bacteria which are not actively causing infection. Consequently, growth does not necessarily reflect infection. Misinterpretation of inappropriately collected cultures results in unintended consequences including unnecessary use of antibiotics, antibiotic resistance, and prolonged hospital stay.

**Methods:**

We created a clinical decision support tool as part of the BrightStar2 initiative using signs of acute respiratory infection with the aim to safely minimize collection of cultures lacking diagnostic utility. We completed a retrospective study of tracheal aspirate cultures (TAC) collected in a 36-bed ICU at a pediatric tertiary care hospital from January to December 2022. We determined whether cultures met support tool criteria for collection, including signs of infection, respiratory change, and lack of previous TAC within 48 hours. We then investigated results of unsupported cultures to determine whether cultures provided clinically actionable information.

**Results:**

Of 200 tracheal aspirate cultures, 133 cultures (67%) did not meet criteria for collection, most commonly due to lack of change in respiratory status. A total of 27 unsupported TACs grew new organisms (20%) of which only one organism had clinical significance, accounting for less than 1% of unsupported cultures.

**Conclusion:**

This data suggests our clinical decision support tool is effective in identifying patients in whom a TAC will not yield useful clinical information.

**Disclosures:**

**All Authors**: No reported disclosures

